# Current challenges and potential solutions to the use of digital health technologies in evidence generation: a narrative review

**DOI:** 10.3389/fdgth.2023.1203945

**Published:** 2023-09-28

**Authors:** Hassan Mumtaz, Muhammad Hamza Riaz, Hanan Wajid, Muhammad Saqib, Muhammad Hamayl Zeeshan, Shaheer Ellahi Khan, Yesha Rajendrabhai Chauhan, Hassan Sohail, Laiba Iman Vohra

**Affiliations:** ^1^ Department of Public Health, Health Services Academy, Islamabad, Pakistan; ^2^ Department of Internal Medicine, Allama Iqbal Medical College, Lahore, Pakistan; ^3^ Department of Internal Medicine, Shalamar Medical & Dental College, Lahore, Pakistan; ^4^ Department of Internal Medicine, Khyber Medical College, Peshawar, Pakistan; ^5^ Department of Internal Medicine, Dow University of Health Sciences, Karachi, Pakistan; ^6^ Department of Internal Medicine, Baroda Medical College, Gujrat, India; ^7^ Department of Medicine, Ziauddin University, Karachi, Pakistan

**Keywords:** socioeconomic disparities in health, telemedicine, artificial intelligence, privacy, pandemics, humans

## Abstract

Digital health is a field that aims to improve patient care through the use of technology, such as telemedicine, mobile health, electronic health records, and artificial intelligence. The aim of this review is to examine the challenges and potential solutions for the implementation and evaluation of digital health technologies. Digital tools are used across the world in different settings. In Australia, the Digital Health Translation and Implementation Program (DHTI) emphasizes the importance of involving stakeholders and addressing infrastructure and training issues for healthcare workers. The WHO's Global Task Force on Digital Health for TB aims to address tuberculosis through digital health innovations. Digital tools are also used in mental health care, but their effectiveness must be evaluated during development. Oncology supportive care uses digital tools for cancer patient intervention and surveillance, but evaluating their effectiveness can be challenging. In the COVID and post-COVID era, digital health solutions must be evaluated based on their technological maturity and size of deployment, as well as the quality of data they provide. To safely and effectively use digital healthcare technology, it is essential to prioritize evaluation using complex systems and evidence-based medical frameworks. To address the challenges of digital health implementation, it is important to prioritize ethical research addressing issues of user consent and addressing socioeconomic disparities in access and effectiveness. It is also important to consider the impact of digital health on health outcomes and the cost-effectiveness of service delivery.

## 1. Introduction

Digital healthcare refers to instruments and services that employ information and communication technologies (ICTs) to enhance and optimize health and lifestyle management, as well as play a role in prevention, diagnosis and treatment of diseases. Since technological competencies have developed and the usage of user-generated digital health data has become more significant in recent years, it may now be used in addition to traditional electronic health data. Additionally, the current shift toward patient-centered, value-based care offers a chance for user-generated digital health data to play a crucial role in medical practices and in the creation of Real-World Evidence (RWE) (1). The US Food and Drug Administration (FDA) views the field of digital health as having a vast reach that encompasses areas including wearable tech, telehealth and telemedicine, mobile health, health information technology, and customized medicine (2). Based on the possible levels of concern to patients, the following categories of digital solutions can be made: approaches that increase system effectiveness but have no discernible impact on patient outcomes, clinical decision support (CDS) and prediction models that direct therapy, provide active monitoring, compute and/or diagnose problems and mobile digital health that informs or delivers basic monitoring, encourages behavior modification and self-management (3). Digital health evidence may be created from digital health data by using computer sciences and other suitable analytical techniques (1). One of the main goals of digital health is the facilitation of data transfer between patients, devices, and physicians. The ideas of customized, preventive, and predictive digital health are strongly connected. With improvements in this connection, information exchange between practitioners and patients can happen in a more timely and intelligent manner (4).

The Digital Health Translation and Implementation Program (DHTI) was established by the Murdoch Children's Research Institute in Australia to incorporate digital technology into healthcare management. The DHTI team, composed of specialists in digital health, researchers, and clinicians, develops hospital-ready solutions to solve challenges in the healthcare system and improve patient care. To assess the usability of the virtual tool model, the DHTI team conducted a series of tests, including semi-structured interviews with healthcare professionals, a survey of individuals in the community, and a survey of parents of children suspected of having concussions. The data collected was used to refine the design of the electronic tool and emphasized the importance of evaluating usability in various contexts (5).

Ayomide Owoyemi and his team (6) conducted a literature review that analyzed the various digital health tools used in various healthcare settings and conditions in Africa. The review paper examined a range of articles and papers on the use of digital tools, the health conditions being addressed, the type of study being conducted, the population involved, the challenges encountered, and the strategies implemented to overcome these challenges in African countries. Many African countries have adopted digital health tools to expand the range of health services offered in hospitals and primary care center. These tools have been highly effective in improving the health of the population by enabling the monitoring of disease outbreaks and surveillance, contact tracing in the event of an infectious disease, the use of electronic medical records, and the provision of services through telemedicine (6). However, certain challenges have emerged in Africa, such as workforce and infrastructure gaps, disparities in digital adoption among the public, low socioeconomic levels, and a lack of electronic infrastructure (7). To address these challenges, it is crucial to evaluate the electronic tools being designed.

The management of healthcare records and remote monitoring has undergone a steady evolution in recent decades. Despite the challenges, it is still important to incorporate key concepts such as computerizing error logs and crash reports in the case of electronic monitoring systems. The Global Task Force on Digital Health for TB was established by the World Health Organization (WHO) with the goal of ending tuberculosis through the development of digital health innovations. Falzon et al. (8) provided insights into digital health products, services, and interventions that are being used globally to improve the management of TB in their paper (8).

Digital health has the potential to revolutionize the way healthcare is delivered, particularly in resource-limited settings. The WHO has outlined the potential benefits of digital health, including increased access to healthcare, improved quality and safety of healthcare, and increased efficiency in healthcare delivery. However, it is important to address the challenges and ethical considerations associated with the implementation of digital health solutions. These challenges include issues related to data security and privacy, the digital divide, and the need for adequate regulation (9).

M. Apro and his team (10) have explored the potential use of e-health tools to assist in the care of cancer patients by tracking their symptoms and managing their treatment. One study found that the use of a web-based therapy significantly reduced tumor symptom distress. A randomized controlled trial (RCT) discovered that a web-based exercise intervention led to significant improvements in overall health status and reductions in pain intensity compared to control groups (10). Magrabi et al. (11) have highlighted the importance of evaluating digital health solutions in the context of the COVID-19 pandemic. They suggest that technologies should be evaluated based on their technological maturity and the size of their deployment and should use the complex systems model to assess the value of data provided by the technology (11).

## 2. Avenues of digital health revolution and challenges

Digital health technologies have the potential to revolutionize the way healthcare is delivered, with the ability to facilitate remote monitoring, improve disease management, and provide more personalized treatment. These technologies include telemedicine, mobile health apps, wearable devices, electronic health records, and artificial intelligence. By gathering data from patients' vital signs, lifestyles, and medical histories, digital health can support the development of a personalized medicine model. However, it is important to carefully evaluate the effectiveness and safety of these technologies before widespread implementation, utilizing both traditional evidence-based approaches and complex systems models (12, 13).

## 3. Utilization of digital health mechanisms

**Figure 1:**
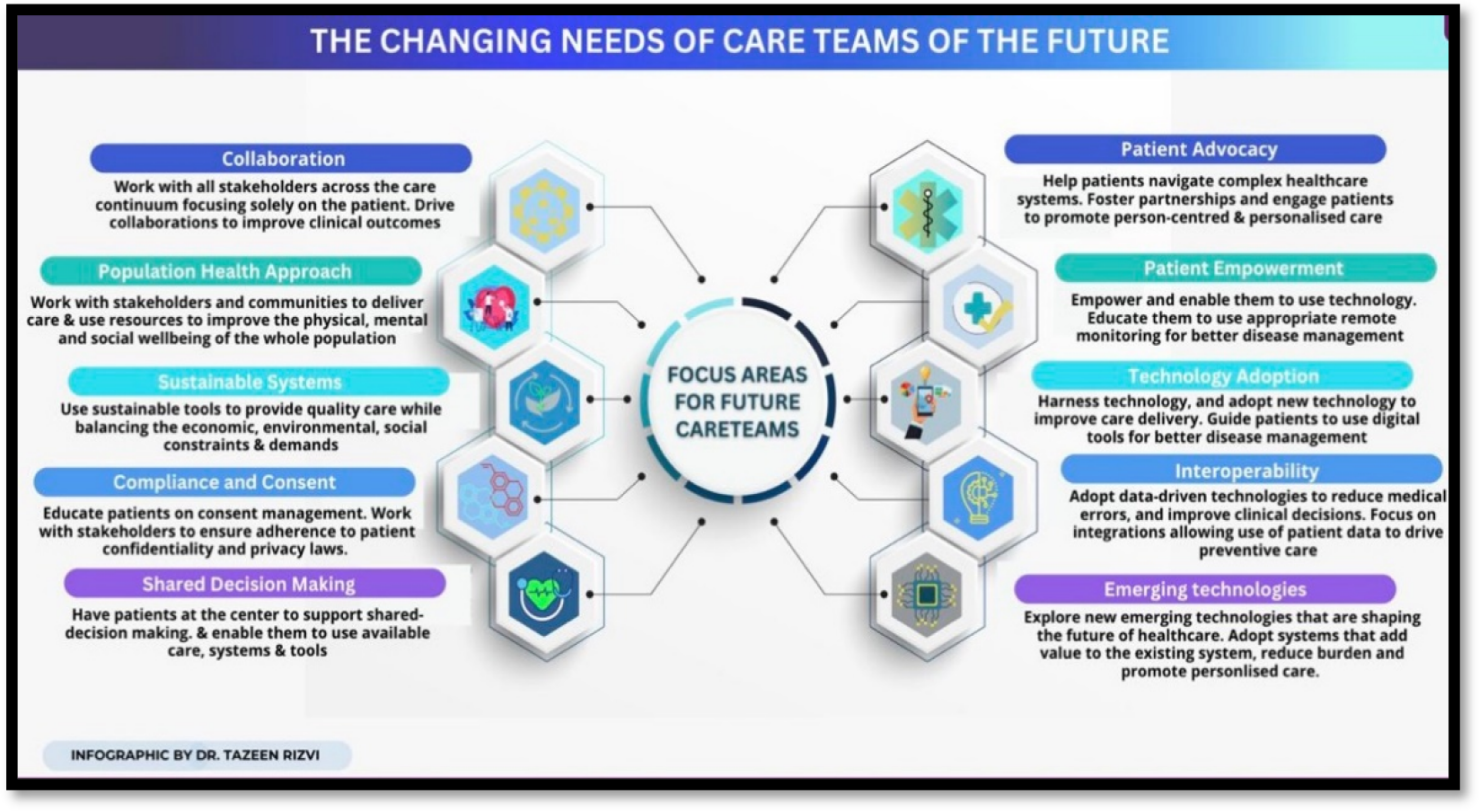
The changing needs of care teams of the future; adopted and published with permission from Dr. Rizvi T.H. (see Rizvi TH, University of Oxford, The changing needs of care teams of the future [Infographic-Internet], 2022, Available from [https://www.linkedin.com/posts/drtazeenrizvi_healthcare-health-wellbeing-activity-7014261135415648256-ouWU?utm_source=share&utm_medium=member_desktop]).

It is important to note that digital health solutions can greatly aid in the fight against COVID-19 and other pandemics. These solutions include telemedicine, mobile health, wearable tech and biosensors, electronic health records, artificial intelligence, and machine learning. These tools can be used for primary care and prevention, screening, monitoring, and surveillance. In addition, social media platforms and Google Trends analyses can be helpful in tracking the spread of the virus and understanding public response. However, it is crucial that healthcare professionals are trained in the use of these digital tools and are involved in the implementation process in order to fully utilize their potential. By prioritizing people and processes over technology, digital health solutions can be effectively used to improve the overall health and wellness of individuals and communities (13, 14). Some ways in which care teams could focus on for future readiness are summarized in Figure 1.

Digital health technology has become a key tool in the fight against COVID-19, with various models being implemented to help with primary care and prevention, screening, monitoring, and surveillance. Telehealth, smartphone apps, and websites are all being used to provide virtual visits, virtual treatment, remote patient monitoring, risk assessment, and triage. In Singapore, the TraceTogether app has been used to monitor COVID-19 infected individuals and notify those who have come into contact with them. In the triage process, individuals are first classified as potentially infected or uninfected before arriving at the hospital, and then undergo rapid clinical evaluation and testing at the hospital to quickly determine their risk level for COVID-19. It is important for healthcare professionals to be trained in the use of these digital tools and be involved in the implementation process in order to fully utilize their benefits and improve the overall health of individuals and communities through proactive and preventive care models (13, 14).

## 4. Challenges faced of using digital health during and post COVID-19

Several challenges impede the forward progress of digital health. These include lack of evidence-based standards, privacy concerns, issues with data governance, and ethical issues among many others (12, 14). One major issue is the sensitivity of health data, which can raise privacy concerns when digitized (14). Governments also face challenges in managing and protecting data (15). Another ethical issue is the issue of consent, as many users may not fully understand the conditions of use when they agree to them (12). Additionally, there is a lack of evidence on the effects of digital health strategies on health outcomes, cost-effectiveness, and system efficiency (16). The effectiveness of telehealth platforms may also be affected by the income and socioeconomic status of users (14). Certain groups, such as minorities, the elderly, and those in low-income or rural areas, may have difficulty understanding and using digital health solutions due to lower health literacy levels (16).

## 5. Opportunities for future use of digihealth

**Figure 2:**
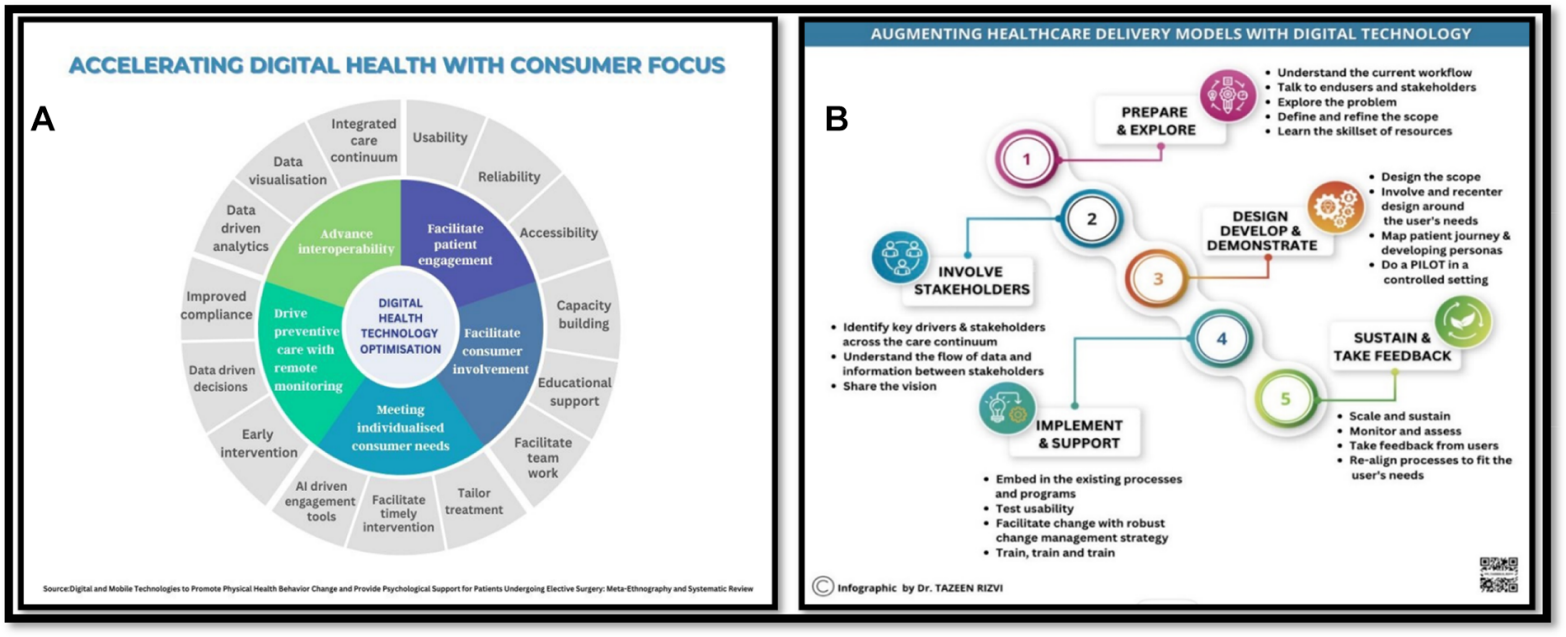
(**A**) accelerating digital health with consumer focus; (**B**) augmenting healthcare delivery models with digital technology; adopted and published with permission from Dr. Rizvi T.H. (see Rizvi TH, University of Oxford, Accelerating digital health with consumer focus [Infographic-Internet], 2022, Available from [https://www.linkedin.com/posts/drtazeenrizvi_digitalising-healthrecords-digitalhealth-activity-7009510993365999617-lxEC?utm_source=share&utm_medium=member_desktop]; Augmenting healthcare delivery models with digital technology [Infographic-Internet], 2022, Available from [https://www.linkedin.com/posts/drtazeenrizvi_accessibility-healthservices-digitalhealth-activity-7004120405506465792-7JdT?utm_source=share&utm_medium=member_desktop]).

It is important to ensure that telehealth becomes a permanent part of national healthcare systems, rather than just being used in emergency situations (13, 16). This would allow for more widespread use of telehealth during epidemics. Investment in 5G technology and the development of modern communication infrastructure in developing countries can also facilitate the implementation of telehealth in these regions (14). Digital health has evolved from simply digitizing medical records to implementing comprehensive information and communication solutions for delivering high-quality healthcare across the care continuum. Companies are using connected sensors, smartphones, diagnostic algorithms, and wearables to capture health data, provide feedback, and facilitate proactive care models. Patient-centric systems that promote patient engagement are becoming more important as consumers become more empowered. Health companies and leaders should embrace new approaches presented by the emerging health ecosystem. An infographic outlining how digital health technology can be used for different purposes along with an infographic depicting augmentation of healthcare delivery models is shown in Figure 2.

## 6. Using digital health technology to better generate evidence and deliver evidence-based care

### 6.1. Current landscape of digital health

#### 6.1.1. Machine learning

Machine learning (ML) is a method that allows computers to process and analyze data more efficiently. It has become more popular as the availability of large datasets has increased. ML has several advantages over traditional statistical techniques, including its flexibility and ability to handle large amounts of data, making it useful for a variety of applications, such as predicting risks, making diagnoses, and forecasting survival rates (17). In the field of medicine, many different algorithms have been used, including for the analysis of optical microscopic malaria detection using ML techniques (18). ML has also been used to predict the occurrence of dementia and Alzheimer's disease by analyzing a range of genetic and environmental factors (19).

#### 6.1.2. Digital apps used for decision support and management

One of the first digital health applications to be approved by the FDA for managing diabetes mellitus was called BlueStar (20). Decision support apps like this allow patients to manage their health beyond clinic visits. The unprecedented proliferation of such tools has led to increased recommendations from national regulatory bodies, such as the draft guidance from the FDA addressing concerns about the use of clinician and patient decision-support systems (21). Software that meets the requirements for a device under the Federal Food, Drug, and Cosmetics Act is subject to FDA regulation. Digital health technologies have the potential to improve health outcomes by increasing patient involvement in self-care and caregiver care, improving communication, and tailoring services to individual needs. In 2018, over $9 billion was invested in digital health startups by venture capital and private equity, an increase of $2 billion from the previous year (22).

#### 6.1.3. To improve research recruitment

Recruiting patients for clinical research can be a significant challenge, especially for randomized controlled trials (RCTs). Poor recruitment can lead to increased costs, longer completion times, or even the termination of a study (23). In a study on boys with Klinefelter syndrome, recruiting children presented unique difficulties. To investigate the effectiveness of traditional and IT-based recruitment methods for reaching hard-to-reach populations such as these, the authors conducted a literature review and compared IT-based outreach to traditional methods such as brochures, flyers, and physician referrals. They found that distributing flyers and brochures and sending letters to doctors and patients at a large hospital and affiliated clinics resulted in only 4 (9%) participants over a three-month period, while web-based tools were more effective at recruiting these individuals. This demonstrates the potential of IT-based tools to improve patient recruitment in clinical research (24).

#### 6.1.4. To obtain informed consent

Informed consent is a process that ensures that research subjects are fully informed about the details of a study and freely choose to participate. This process is governed by ethical standards and laws to protect the rights of human subjects. The goal of informed consent is to provide potential participants with sufficient information in a clear and understandable form so they can make an informed decision about whether or not to participate in the research. Informed consent is a crucial aspect of patient engagement, but as the process has become more formalized, it has also created some challenges. To address these challenges, innovative methods such as video consent, smartphone consent, and digital informed consent are being used (25). For example, the Patient and Provider Assessment of Cholesterol Management (PALM) study used a tablet-based platform to gather patient-reported data on behaviors and attitudes related to lowering cardiovascular risk and managing lipid levels. The study also surveyed healthcare providers about their attitudes towards existing lipid-lowering targets and barriers to optimal cardiovascular risk reduction (26).

#### 6.1.5. Digital formularies

A formulary is a list of drugs that is used by a purchaser, pharmacy, or provider organization to identify which medications are preferred or not based on factors such as price and therapeutic value (27). In some cases, formularies specify which drugs can be prescribed, while in other situations, formularies are used to negotiate reduced pricing, such as for a specific proton-pump inhibitor. With the large number of health apps available, a “digital formulary” for apps that covers a range of applications, including monitoring, diagnosis, and therapeutics, could be a useful tool for incorporating apps into clinical treatment. Recently, Express Scripts, a major US pharmacy benefits management company, unveiled a digital health formulary which could prove to be a significantly useful tool in clinical care in the future (22).

### 6.2. Potential problems with digital health care

Digital health innovations have the potential to address various challenges faced by healthcare systems, such as barriers to engagement, resource constraints, and unmet patient needs. However, there are also challenges to implementing digital health solutions, including poor coordination, inadequate stakeholder involvement, weak health systems, and lack of interoperability. To overcome these barriers, it is important to adopt a problem-centered approach, involve all relevant stakeholders, budget accurately, standardize processes and resources, establish an evaluation framework, and provide training and support to users throughout the transformation process.

#### 6.2.1. Health data disparity

Health data disparities refer to differences in the quantity, quality, or both of health data among different people, groups, or populations. These discrepancies can occur within communities as well as across various demographics, fields of study, and medical conditions. For example, in genome-wide association studies, as of 2018, 78% of participants were European, 10% were Asian, 2% were African, 1% were Hispanic, and less than 1% were from any other ethnic group, resulting in a bias towards European genetics in human analysis (28).

#### 6.2.2. Quality of data

Maintaining the quality and reliability of data is a continuous challenge. “Big data” refers to the use of humongous amounts of patient-related data, such as electronic health records (EHRs) or other sources, to understand population risk, treatment outcomes, and trends in healthcare utilization (29).

#### 6.2.3. Productivity paradox

The productivity paradox, also known as the lack of productivity improvements resulting from advances in technology and increased computing capabilities, has been observed in industries other than healthcare. Inefficient management of information technology, a lack of infrastructure to support modern software, and poor technology usability are the main contributors to this issue (30).

#### 6.2.4. Privacy

Data breaches at major companies such as Amazon, Sony, and Equifax have raised concerns about the security of sensitive data stored in large systems. Digital health also needs significant improvements in data security, as demonstrated by recent instances of health-related data hacking, which have included ransom attacks on clinic computer networks and unauthorized remote modification of defibrillators (31).

### 6.3. Future implications of digital health

#### 6.3.1. Randomized clinical trials

Digital health technology has the potential to facilitate and improve traditional RCTs, which are becoming increasingly expensive and complex, and take a long time to complete and implement (23). Although digital technologies have not been utilized to the maximum efficiency and data regarding integration of these technologies to clinical trials haven't been examined in detail, it is still important to assess these technologies in order to conduct more efficient and practical studies. Once issues such as accuracy, reliability, access, confidentiality, and the need for regulations have been addressed, the potential benefits of digital health technology for clinical utility in patient-care and research purposes can be better understood. However, it is also important to consider potential negative effects such as a paradoxical loss of productivity, decreased quality of care, and other undesirable outcomes of incorporating digital technologies into clinical pathways (32).

#### 6.3.2. Ambivalence in digital health

The concept of “ambivalence” has recently been proposed as a way to understand the complexities and nuances at play when digital technologies are integrated into care practices, in response to polarized views on the pros and cons of digital health. In a study of 97 HIV patients' interviews and co-design workshops, Marent et al. (33) argue that a relational and multifaceted understanding of ambivalence can provide a nuanced understanding of the multiple impacts and social enactments of digital health. This suggests that implications and reactions vary across dimensions and within specific practices, rather than being uniformly positive or negative. For example, cross-dimensional ambivalence means that an HIV patient may appreciate certain features of a digital device (such as timely reminders to take medication) while disliking others (such as access to blood test results, which can bring uncertainty) (33).

### 6.4. Improving digital health care: Way forward

One of the key considerations in providing digital health is understanding the needs and perspective of patients. There can be many challenges and difficulties in integrating digital health into daily life, such as concerns about data security and privacy, and a lack of alignment with patients' needs and values. To address these issues, it is important to develop robust systems with strong data security and privacy, and to engage with technology developers and patients early on to ensure that digital innovations address patients' concerns. It is also essential to involve healthcare professionals and adapt healthcare systems to the use of digital health. In healthcare systems, the current infrastructure may not be able to accommodate the integration of new digital health technologies, and there may be concerns about reimbursement for these services. To address these challenges, it is necessary to conduct rapid pilot experiments with existing healthcare infrastructure to develop systems that can adapt to digital health technologies, and to engage with clinical researchers to ensure that digital technologies being tested are cost-effective and can be effectively integrated into the digital healthcare infrastructure and systems. Many digital health solutions are still in their early stages and will need time to evolve and be validated for clinical accuracy. Improving the relationship between researchers, scientists, clinicians, regulators, and technology developers can help achieve the ultimate goal of providing better healthcare and support for patients.

#### 6.4.1. Limitations

Digital health technologies utilized for evidence-based medicine, such as telerehabilitation, face several limitations. One key barrier is the reliance on hands-on approaches in healthcare, such as manual therapy and acupuncture, which are difficult to replicate remotely. To overcome this, a hybrid model combining online sessions with in-person visits for hands-on techniques can be implemented. Additionally, the quality of health information available online is often non-scientific, biased, and not aligned with evidence-based practices. This can mislead patients and create unrealistic expectations. Limited digital health literacy among certain social groups, resistance to change among healthcare professionals, technological issues, and resource constraints also pose challenges. Communication and trust-building in the digital environment, as well as infrastructure and public policy considerations, further impact the adoption and effectiveness of digital health technologies. Overcoming these limitations is crucial to ensure equitable access, quality care, and patient engagement in digital health interventions (34). The lack of standardized data-sharing agreements and transactional standards between institutions hinders the infrastructure needed for effective data sharing and integration in public health. The COVID-19 pandemic has highlighted the importance of data sharing and ethical considerations in the field of digital health. Challenges include maintaining control over generated data while promoting their use for scientific discoveries and obtaining informed consent for data collection and access. Limited resource capacity, including infrastructure and internet access, poses challenges for implementing and evaluating digital health technologies, particularly in resource-limited settings. Digital health literacy among the general population plays a crucial role in the acceptability and adaptability of digital health solutions. The shortage of qualified professionals in digital health further impedes the widespread adoption of digital health applications. Insufficient policies, governance structures, standard operating procedures, and financial resources hinder the successful deployment of digital health interventions. Culturally adapted technologies and careful consideration of the local context are necessary for effective implementation. The transition from face-to-face care to digital health-enabled remote care varies in its advantages and disadvantages depending on the country, program, and specific digital health technology employed (35).

While this review article offers valuable insights into the utilization of digital health technology, it is important to acknowledge a few limitations. Firstly, the scope of the article is focused on specific aspects of digital health, such as machine learning, decision support apps, research recruitment, informed consent, and digital formularies. While these topics provide a solid foundation for understanding the current landscape, it is possible that other important aspects of digital health may not have been extensively covered. Secondly, as the field of digital health is evolving rapidly, there is a chance that some of the information presented in this article may become outdated over time. It is crucial to stay abreast of the latest developments in the field and consider incorporating newer innovations or trends into future revisions. Despite these limitations, this review article offers valuable insights into the potential of digital health technology and serves as a foundation for further exploration and research in this exciting and rapidly evolving field.
